# The Impact of Thermocycling Process on the Dislodgement Force of Different Endodontic Cements

**DOI:** 10.1155/2013/317185

**Published:** 2013-08-24

**Authors:** Mohammad Ali Saghiri, Armen Asatourian, Franklin Garcia-Godoy, James L. Gutmann, Nader Sheibani

**Affiliations:** ^1^Department of Ophthalmology and Visual Sciences, University of Wisconsin School of Medicine and Public Health, Madison, WI, USA; ^2^Kamal Asgar Research Center (KARC), Iran; ^3^Bioscience Research Center, College of Dentistry, The University of Tennessee Health Science Center, Memphis, TN, USA; ^4^Department of Restorative Sciences, Texas A&M University Baylor College of Dentistry, Dallas, TX, USA

## Abstract

To evaluate the effects of thermocycling (500 cycles, 5°C/55°C) on the push-out bond strength of calcium silicate based cements including WMTA, Nano-WMTA, and Bioaggregate to root dentin. Forty-eight dentin slices were prepared and divided into 3 groups (*n* = 16) and filled with Angelus WMTA, Nano-WMTA, or Bioaggregate. After incubation, half of the samples were thermocycled while the other half remained untreated. Push-out bond strength was calculated, and the modes of the bond failures were determined by SEM. The highest bond strength was seen in nonthermocycled Nano-WMTA samples and the lowest in thermocycled Bioaggregate samples. The significant differences between nonthermocycled and thermocycled samples were only noticed in WMTA and Nano-WMTA groups (*P* < 0.001). The mode of failure for thermocycled samples of all three cements was mostly cohesive. Thermocycling process can drastically affect the push-out bond strength of calcium silicate based cements. The intrastructural damages occurred due to the thermal stresses, causing cohesive failures in set materials. Sealing property of endodontic cements which have experienced the thermal stresses can be jeopardized due to occlusal forces happening in furcation cites.

## 1. Introduction

One of the most crucial reasons for the failure of endodontically treated teeth is the leakage of applied endodontic materials, which is indicated as a failure in obstructing the communications between root canal structure and its nearby tissues [1, 2]. Good sealing property has been introduced as a prominent characteristic of an ideal root-end filling and repair material which can guarantee its longevity and increase the success rate at the end [3]. Mineral trioxide aggregate (MTA) due to its biocompatibility, bioactivity, nontoxicity, radio opacity, dimensional stability, insolubility, and especially for its unique superior sealing ability has gained wide spread acceptance among clinicians through many years [4]. Direct and indirect pulp capping, pulpotomy, treatment of teeth with immature root formation, handling root perforations, and root-end filling surgeries are some of the applications of this desirable cement [5]. However, authors have mentioned some drawbacks for MTA including slow setting time, granulated constancy, porosity, and decreased sealing ability in low pH environments occurring as a result of the reduction in surface hardness that can cause lower push-out bond strength [6, 7]. It has been also revealed that the push-out bond strength of MTA declined because of some factors such as pH, final root canal treatments, and the humidity of the environment [7–9].

Two new cements have been presented with the aim of enhancing some of the MTA shortcomings. The first cement is Bioaggregate (Innovative Bioceramix, Vancouver, BC, Canada), an innovative endodontic filling and repair cement which is mainly composed of calcium silicate, calcium hydroxide, and hydroxyapatite [10] and acclaimed to be similar to MTA from the aspect of biocompatibility. It is aluminium free and contains tantalum pentoxide for the opacity, instead of bismuth oxide in MTA [10, 11]. Nano-WMTA is another version of MTA which is a nanomodified composition of WMTA (Kamal Asgar Research Centre), acclaimed to possess nanoparticle size powder that increases the contact surface area and accelerates and enhances the hydration process [12]. Authors have shown that, by reducing the particle size and adding some substances such as tricalcium aluminate, calcium sulfate, zeolite, and strontium carbonate some drawbacks such as long setting time and decreased resistance in low pH can be fulfilled [13]. It was indicated that, by making structural changes mentioned before, the push-out bond strength of the cement can be improved against the dislodgement forces [8]. 

In previously done studies, many authors have questioned the effect of temperature changes on the Portland cement [14, 15]. It was mentioned that this cement material can be affected by the thermal changes occurring after its setting time [15]. Due to these thermal stresses microstructural changes can take place which resulted in remarkable changes in physical properties of the set material [15, 16]. Thermocycling is a laboratory method of exposing dental materials and tooth to temperature ranges similar to those occurring in the oral cavity that could produce adverse consequences as a result of different coefficients of thermal expansion between the tooth structure and the filling material. Through these cycles, thermal stresses could affect the bond strength between the repairing or filling material and the tooth structure [16–19]. Some authors have questioned the undesirable influence of thermal loads on dental materials which are used inside the root canal. It was reported that thermocycling might cause drastic effects on the bond strength of these materials which can increase microleakage and dentin bond failure [20, 21].

The present study was carried out to evaluate the effect of thermocycling process on the bond strength of WMTA, Nano WMTA, and Bioaggregate to root canal dentin. The hypothesis tested whether the in vitro thermal changes, which can resemble the thermal stresses happening in the oral cavity, can affect the push-out bond strength of these filling and calcium silicate based cements. The negative impact of this process might influence the sealing characteristics of these cements and increase the possibility of leakage that ultimately results in treatment failure in clinic.

## 2. Materials and Methods

Forty-eight extracted single-rooted human teeth were selected and, after decoronation, were sectioned horizontally by using a water-cooled low-speed IsoMet diamond saw (Buehler, Lake Bluff, NY, USA) at the midroot part to prepare 2 mm thickness dentin slices. The samples canals were enlarged by using number 2 through number 5 Gates-Glidden burs (Mani, Tochigi, Japan) to form 1.3 mm diameter standardized spaces. Prepared slices were first immersed in 17% EDTA for 1 minute, then in 1% sodium hypochlorite for the same period of time. After immersion, sections were washed in distilled water, dried immediately, and randomly divided into three groups of sixteen in each (*n* = 16). Three types of cement were mixed according to manufacturer instruction and placed inside the canal spaces by using conventional technique as follows:

 group A: WMTA Angelus (Angelus Dental Industry Products, Londrina, Brazil);
 group B: nanomodification of WMTA (Kamal Asgar Research Centre) [12];
 group C: Bioaggregate (Innovative BioCeramix, Vancouver, BC, Canada) [10].

Dentin slices were covered by pieces of gauze soaked in synthetic tissue fluid (STF), which was prepared as follows: 1.7 g of KH_2_PO_4_, 11.8 g of Na_2_HPO_4_, 80.0 g of NaCl, and 2.0 g of KCl in 10 L of H_2_O (pH 7.4) and incubated in 37°C, 98% humidity for 3 days. It should be noted that the pieces of gauze were refreshed every 6 hours to maintain the stable media. Prior to any further treatment, samples were probed with an explorer to ensure all cement materials were completely set and solid. Thereafter, each experimental group was subdivided into two subgroups of eight in each (*n* = 8). The first subgroup specimens of each group did not receive any treatment and served as control, while the second subgroup samples were subjected to thermocycling process in water between 5°C and 55°C for 500 cycles. The storage time in each bath was 20 seconds, and the transfer time between the two baths was 5 seconds. All samples were immediately subjected to push-out bond strength testing.

### 2.1. Push-Out  Test

The universal testing machine Zwick/Roell Z050 (Ulm, Germany) was used for the push-out bond strength evaluation of samples. Each specimen was placed on a metal slab with a central hole to allow the free motion of the plunger. A compressive load was applied to the surface of WMTA, Nano WMTA, and Bioaggregate samples by the downward pressure motion of 1.00 mm diameter cylindrical stainless steel plunger at a crosshead speed of 1 mm/min. The clearance of plunger was set approximately 0.2 mm from the margin of the canal wall to ensure the plunger would apply the loads only on the surface of the cements. The maximum load applied to cement plug at the time of dislodgement was recorded in newton, and the megapascal, (MPa) values were calculated by the following formula:

(1) $$\begin{array}{c} \text{Debond}\;\text{stress}\;\;\left(\text{MPa}\right)=\frac{\text{Debonding}\;\text{force}\;\;\left(\text{N}\right)}{\text{Surface}\;\text{area}\;\;\left({\text{mm}}^{2}\right)}. \end{array}$$

### 2.2. SEM and Stereomicroscope Analysis

 Two samples from each subgroup were observed under a scanning electron microscope (Cambridge Instrument Leo 440 *i*, England) at ×1000 magnification. Also the fractured specimens of each group were examined under a stereomicroscope equipped by a digital camera attached to the stereomicroscope (Olympus, SZM9) with ×16 magnifications, and the type of bond failure (adhesive, cohesive, or mixed) was recorded. Bond failure was characterized according to the area of cement remaining on the dentin surface and categorized as the following:

 adhesive failures: less than 25 percent of cement remnant on the root canal dentine;
 cohesive failures: greater than or equal to 75 percent of cement remnant on the root canal dentin;
 mixed adhesive/cohesive failures: 25 to 75 percent of cement remnant on the root canal dentin.

The data for each material was analyzed using analysis of variance (Anova), and post hoc comparisons were performed using Tukey-B. The level of significance was 0.05. 

## 3. Results

**Figure 1:**
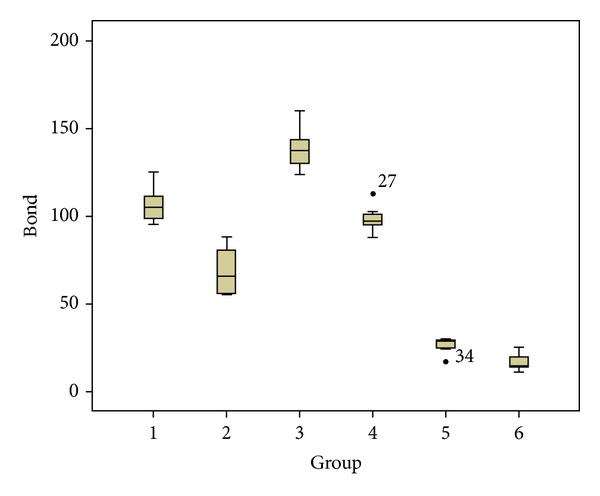
The box plots of means and standard deviations of push-out bond strength values of experimental groups. (1) Untreated WMTA, (2) thermocycled WMTA, (3) untreated Nano-WMTA, (4) thermocycled Nano-WMTA, (5) untreated Bioaggregate, and (6) thermocycled Bioaggregate.

 The means and standard deviations of push-out bond strength values of the both thermocycled and nonthermocycled subgroups of WMTA, Nano-WMTA, and Bioaggregate are presented in Figure 1. Statistical analysis showed significant differences between thermocycled and nonthermocycled samples in WMTA and Nano-WMTA groups (*P* < 0.001), while the difference was not significant between thermocycled and nonthermocycled subgroups of Bioaggregate (*P* = 0.247).

The highest value was recorded in nonthermocycled Nano-WMTA samples and the lowest in thermocycled Bioaggregate samples. The comparison among subgroups of all three experimental groups indicated that significant differences were seen between all subgroups (*P* < 0.001) except for the nonthermocycled WMTA samples and thermocycled Nano-WMTA specimens (*P* = 0.529) (Figure 1, 1–4). 

The samples observation under stereomicroscope with ×16 magnifications has shown the modes of bond failure in untreated and thermocycled samples of each experimental group. According to the observation, the majority of failure modes were cohesive in all three experimental groups, which are indicated in detail in Table 1. 

## 4. Discussion

 Push-out bond strength is one of the most important in vitro characteristics that can be extended to a clinical situation and translated to sealing and retentive ability of a root-end filling material and repairing perforation cement during clinical practices [4]. This test is an effective, dependable, and feasible method to evaluate the stability of an endodontic material in its surrounding root canal dentin [7, 8]. Previous authors have mentioned that Portland-based cements such as MTA consist of dicalcium and tricalcium silicate [22]. It was pointed out that dicalcium silicate in comparison with tricalcium silicate needs more time for hydration which makes MTA require a wet environment in order to gain its optimal physical properties [4, 23]. In accordance with this fact, in the present study all cement materials have been incubated in condition with 98% humidity for 3 days to ensure that the hydration process was completed in set materials. The 2 mm thickness of dentin slices was selected according to the previously done investigations on the push-out bond strength of MTA [7–9], in which some of them have used even 1 mm thickness of dentin slices [7, 8]. However, it should be mentioned that some authors have pointed out that the effective barrier thickness of this cement is 4 mm [24]. 

 Thermocycling process has been introduced as an artificial aging methodology [25] which is utilized for evaluating the influence of thermal stresses on the bond strength of dental materials. By the means of these thermal cycles in in vitro situation, the impact of different coefficients of thermal expansion for these restorative and repair materials can be investigated [16–19]. Through previous studies, authors have used this method for materials such as adhesive systems, two-step bonding systems, luting agents, nanocomposites, and zirconia ceramics [16–20]. Investigators have shown that thermocycling regimen comprising a minimum of 500 cycles in water between 5°C and 55°C is an appropriate artificial aging test [25]. This issue was also taken into granted through present study, and all three tested cement materials were subjected for these thermal cycles in order to analyze the impact of thermal shocks or stresses on the bond strength of cements to root canal dentin. 

 The findings of this study have indicated that untreated samples of Bioaggregate group showed lower push-out bond strength in comparison with the untreated MTA and Nano-WMTA specimens. In previous investigations, similar results have been reported as Bioaggregate has exhibited lower values of push-out strength after 34 days in comparison with MTA [26]. This result was also consistent with the findings of Saghiri et al. where these authors have questioned the push-out bond strength of Nano WMTA, MTA, and Bioaggregate and reported that Nano-WMTA along with MTA showed superior property in comparison with Bioaggregate [8]. One of constituents of Bioaggregate is amorphous silicon dioxide [10], which is demonstrated to play an important role in further reaction of calcium hydroxide (CH) to form C-S-H that resulted in reduced amount of CH in set material [27] and ultimately weakened the cement structure against dislodgement forces [28]. According to previous investigations, Nano-WMTA can provide more contact surface area and better hydration which promotes the physical properties of this cement in comparison with Bioaggregate and MTA [8]. On the other hand, additives such as zeolite which is a crystalline hydrated aluminosilicate of alkaline metals and metals of alkaline soils (Ca, K, Na, and Mg) can act as an additive stabilizer agent in Nano-WMTA cement. This substance also has an anticorrosive behavior against sulfate which is responsible for sulfate attack occurring through the reaction of Portland cement [29]. This issue can explain the significant differences between the push-out bond strength values of Nano-WMTA with MTA and Bioaggregate, respectively. It should also be mentioned that although the thermocycled and nonthermocycled samples of Bioaggregate cement showed the lowest push-out bond strength values in comparison with other two tested cement samples, the difference between thermocycled and nonthermocycled specimens was not significant in case of Bioaggregate and which was not detected in WMTA and Nano-WMTA cements. This issue might suggest some resistance by Bioaggregate cement against the thermocycling effects on cement structure; however, the push-out bond strength values of WMTA and Nano WMTA samples after thermocycling were more than Bioaggregate cement results.

 The results of thermocycled samples in all three experimental groups showed that thermocycling process had significantly decreased the push-out bond strength of these samples in comparison with untreated specimens. These outcomes are consistent with previously done studies where authors have tested the effect of this process on the bond strength of other dental materials such as bonding systems or composites [16, 17, 19]. Previous investigators have mentioned that thermocycling process consists of thermal changes accompanied by additional exposure to water. Thermal stresses generated inside the filling or repair materials can produce thermal expansions which can negatively affect the interface of these materials and the tooth structure [30]. 

 The SEM and stereomicroscope observations have revealed that the type of bond failure in untreated samples of MTA and Bioaggregate was mostly cohesive with some cases of adhesive or mixed patterns. This result was inconsistent with previous studies where the adhesive type of failure for MTA was mentioned [7, 31]. This difference might be explained by the short storage time (3 days) in the present study comparing with previously mentioned studies where authors used 4 and 7 days [7, 31] which definitely can affect the hydration process [4, 23]. Nonthermocycled Nano-WMTA samples showed cohesive failure modes for the majority of cases which is consistent with previous study [8]. This type of bond failure can be attributed to the nanoscale particles which are responsible for intimate contact of powder particles that resulted in interlocked pattern and more possibility for cohesive mode of failure. The observation of thermocycled samples of all three groups surprisingly showed more cohesive failure modes (Table 1). This outcome is along with the results of decreased push-out strength which were seen in the present study. Detected cohesive modes of failure can suggest that thermal stresses as well as the water bath cause structural changes inside the experimented cements [30, 32], which can adversely influence the cement's dislodgement resistance and sealing properties.

 In the literature of Portland cements, many studies have addressed the structural changes of this type of cement after exposure to temperature changes [14, 15, 33]. Authors have mentioned that the temperature rise more than 50°C can lead to unfavorable structural changes in Portland cements which contains Al_2_O_3_/SO_3_ ratio of <1.3 (bulk weight) [15]. In these types of Portland cements small amounts of ettringite are present in temperature more than 50°C, which is claimed to be responsible for the negative changes in physical properties such as compressive strength of these cements [15]. However, this ratio for WMTA [34], Nano-WMTA [8], and Bioaggregate [10] is more than 1.3, which in these types of Portland cements with Al_2_O_3_/SO_3_ ratio of >1.3 even better hydration can occur [14, 15]. Meanwhile, another investigator has discussed the theory of moisture distribution in water-soaked hardened Portland cement [33]. It was reported that the temperature changes can cause shrinkage or swelling in cement structure, which resulted in dimensional changes in the set material [33]. These dimensional changes might explain the decreased push-out values in samples after thermocycling process and also the increased cohesive mode of failure observed in SEM images as well. 

## 5. Conclusions

According to the results of the present study the following could be concluded.

**Figure 2:**
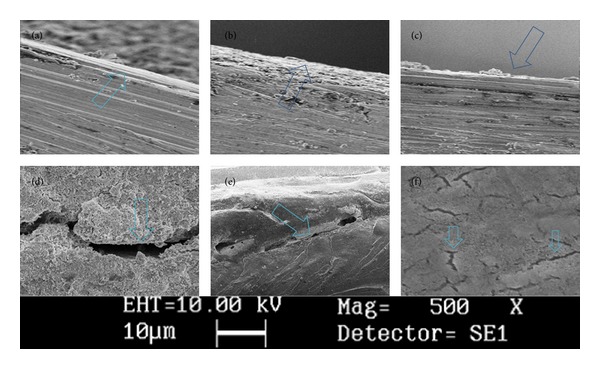
The SEM images of experimental groups after thermocycling in high magnification verify that a thin layer of cement covers the root canal dentin. (a) WMTA, (b) nano-MTA, (c) Bioaggregate. (×1000). WMTA group after thermocycling in low magnification (d) (crack growth). (e) Deboning. (f) Crack initiation over the texture (×500).

- Thermocycling (the ISO TR 11450 standard, 1994) can adversely affect the push-out bond strength of all tested root-end filling materials.
- According to the SEM images shown in Figure 2, the structural changes made by the thermal stresses from thermocycling process can produce undesirable damages that can meaningfully decline the dislodgement resistance of these materials.
- Nano-WMTA and WMTA showed encouraging results; however, thermal changes can significantly increase their cohesive failure which might have negative impact on the sealing characteristics of these cements, especially in the presence of dislodgement forces such as occlusal forces in clinical situation.
- Although the push-out bond strength values of thermocycled and nonthermocycled samples of Bioaggregate cement were lower than WMTA and Nano WMTA, Bioaggregate was the only cement which after thermocycling did not show any significant decrease in push-out bond strength after thermal changes made by thermocycling process. This issue can suggest that Bioaggregate cement has some degree of resistance against the negative effects of thermocycling process.

## Figures and Tables

**Table 1:** Modes of bond failure of untreated and thermocycled samples of each experimental group.

Type of cement	Failure modes in dentin-cement interface			
		Adhesive	Cohesive	Mix
WMTA	Untreated	2	5	1
	Thermocycled	0	8	0
Nano-WMTA	Untreated	0	7	1
	Thermocycled	0	8	0
Bioaggregate	Untreated	1	5	2
	Thermocycled	0	7	1
